# Policy tools, objectives, and governance in China’s public sports services: an X–Y–Z framework analysis of national and provincial policies

**DOI:** 10.3389/fpubh.2025.1692238

**Published:** 2025-10-28

**Authors:** Wenzhe Zhou

**Affiliations:** School of Public Administration, South China University of Technology, Guangzhou, Guangdong, China

**Keywords:** public sports service policy, content analysis, semantic network, public health, X-Y-Z, optimization strategy

## Abstract

**Background:**

As part of “Healthy China 2030” strategy, China has emphasized the expansion and equalization of public sports services to improve population health and promote social equity. Despite these goals, current policies often exhibit fragmented tool selection, vague targeting, and weak alignment with public needs, limiting their effectiveness in supporting inclusive, high-quality development.

**Methods:**

This study proposes an “X–Y–Z” analytical framework that integrates policy tools (X), policy objectives (Y), and thematic features (Z). Using content analysis, 76 national and provincial policy documents (2015–2025) were systematically coded. NVivo14 was used to extract and classify policy tools and objectives, while ROST-CM6 identified high-frequency keywords and constructed a semantic network.

**Results:**

The findings show a heavy reliance on supply-oriented tools, with demand-oriented tools significantly underutilized. Policy objectives are mainly concentrated on industrial development and national fitness, with relatively low attention to equity and economic benefit. Thematic analysis highlights “health,” “construction,” and “development” as core priorities, while “innovation” remains marginalized.

**Conclusion:**

By combining text encoding with semantic network analysis, this study provides a replicable and data driven approach to evaluating policies in the field of public health. It points out structural imbalances and reveals key blind spots in policy design. The research results provide an empirical basis for optimizing tool selection, coordinating multiple objectives, and enhancing policy responsiveness and fairness.

## 1 Introduction

Public sports services are a central component of China’s “Healthy China 2030” strategy, which seeks to improve population health and strengthen social equity. The Healthy China 2030 Planning Outline calls for “substantially increasing the accessibility and coverage of public sports facilities” by 2030, targeting a national fitness participation rate above 40% (1). The National Fitness Program (2021–2025) further promotes the construction of “15-min fitness circles” in urban and rural communities, aiming for at least 38.5% of the population to engage in regular physical exercise by 2025 (2). Despite these ambitious policy goals, recent survey data reveal persistent disparities. Bulletin of the Fifth National Physical Fitness Monitoring indicated that while national fitness levels are gradually improving, the gap between urban and rural populations remains significant, particularly among children and older adults (3). Unequal resource allocation, insufficient rural infrastructure, and limited professional support continue to undermine service equity and public satisfaction.

In response, both national and provincial governments have issued a series of policies to expand facility supply, improve accessibility, and foster cross-sector collaboration (4, 5). However, the effectiveness of these policies is often constrained by fragmented governance, ambiguous policy targets, and insufficient mechanisms for monitoring and evaluation (6). Existing research has mostly focused on qualitative analysis of policy texts, governance models, and the historical evolution of public sports services (7, 8). Comprehensive, quantitative investigations that systematically assess policy design, tool usage, and outcome orientation are still scarce.

To address these gaps, this study develops a three-dimensional “X–Y–Z” analytical framework based on policy tool theory and applies it to 76 national and provincial policy documents. Through systematic content analysis using NVivo 14 and ROST-CM6, the study aims to: (1) identify the structural features and main areas of emphasis in current public sports policies; (2) reveal design preferences and shortcomings; and (3) provide evidence-based recommendations for optimizing tool selection and aligning policy objectives. The findings are expected to contribute to the continuous improvement of China’s public services and support the realization of the “Healthy China 2030” strategy.

## 2 Literature review

Public sports service policy is an important field within the research on public administration and health governance, especially under the strategic backdrop of “Healthy China 2030” (1). Existing studies have affirmed the significant role of public sports services in promoting population health, advancing social equity, and fostering national fitness (9). In the early stage, the research focus was largely on government-led models, emphasizing the state’s dominant position in infrastructure investment and direct service supply (10). While such models have achieved remarkable progress in expanding the coverage of public sports facilities, they have also resulted in a notable urban–rural divide. According to the China Physical Fitness Surveillance Report (2020), the regular participation rate in physical activity is 46.1% in urban areas and only 30.1% in rural areas, with significant disparities persisting among different social groups (11).

To address these challenges, recent research has paid increasing attention to multi-actor and collaborative governance frameworks, recognizing the importance of engaging not only government agencies but also social organizations and market entities in the delivery of public sports services (12, 13). This paradigm shift is in line with policy developments such as the National Fitness Program (2021–2025), which encourages the construction of “15-min fitness circles” and cross-sectoral cooperation to meet the diversified needs of different population groups (14). However, even as the policy system continues to expand, resource allocation imbalances, quality variation, and insufficient professional support still impede the achievement of national goals such as equitable access and universal participation (15–17).

In terms of research methodology, most prior studies have adopted qualitative approaches, including policy text analysis (18), case studies (19), and expert interviews, to explore the structure and effectiveness of public sports service policy. While these approaches offer valuable insights into policy intent and governance logic, the lack of systematic quantitative evaluation restricts the ability to compare policy impact across regions and identify best practices. Only a few studies have attempted to apply policy tool theory or develop comprehensive indicator frameworks for empirical assessment, which limits the practical guidance that research can offer for future policy optimization (20, 21).

In summary, while the literature provides a strong foundation for understanding the evolution and key challenges of China’s public sports service policies, notable gaps remain. Most existing studies rely on qualitative methodologies such as document interpretation, case studies, and expert interviews, which although insightful lack empirical rigor for cross-regional comparison and structural validation. Few attempts have been made to operationalize policy tool theory through systematic coding or to establish replicable frameworks for quantitative assessment. This methodological limitation restricts the ability to generalize findings, evaluate policy impact objectively, or inform data-driven policy optimization. In response, this study offers a novel contribution by developing an “X–Y–Z” analytical framework and applying a structured content analysis using NVivo14 and ROST-CM6 to 76 policy documents. This mixed-method approach integrates quantitative coding, frequency analysis, and semantic network mapping, thereby enabling a more granular, comparable, and reproducible assessment of policy tool usage, objective alignment, and thematic priorities. By moving beyond descriptive or interpretive analysis, the study contributes new methodological tools and empirical insights that can inform both academic research and evidence-based policymaking within the broader framework of “Healthy China 2030” and rural revitalization.

## 3 Data sources and research methodology

### 3.1 Policy document collection and screening

In this study, public sports service policy texts were systematically retrieved from authoritative government platforms, including the official website of the State Council of the People’s Republic of China, the Peking University Law Database, and the General Administration of Sport of China. The retrieval process utilized keywords such as “public sports service,” “national fitness,” and “sports facility construction.” Initially, a total of 112 policy documents issued between 2015 and 2025 at the national and provincial levels were identified. To ensure the accuracy, completeness, and comparability of the sample, policies that were outdated, irrelevant, or classified as “standard” or “approval” notices were excluded. After a rigorous screening process, 76 policy texts directly related to the development and implementation of public sports services were included in the final analysis (see Table 1).

**Table 1:** Name of public sports service policy.

Number	Name of policy document
1	Outline of the “Healthy China 2030” Plan
2	Notice of The State Council on Printing and Distributing the National Fitness Program (2021–2025)
3	Opinions of The State Council on Implementing the Healthy China Initiative
…	…
75	Notice of the Jiangsu Provincial Department of Sports on Issuing the “Jiangsu Province Public Sports Service System Construction Plan (2016–2020)
76	Notice of the Shaanxi Provincial Department of Sports and the Shaanxi Provincial Department of Finance on Issuing the Interim Measures for the Government’s Purchase of Public Sports Services from Social Forces

The 2015–2025 timeframe was selected to align with key national strategies, notably the launch of Healthy China 2030 and the implementation of the National Fitness Program (2021–2025). This period captures a full decade of intensified policy activity in public sports services. However, the analysis is limited to officially published national and provincial-level documents, excluding earlier policies and local implementation texts, which may also contain relevant insights. As a result, the findings may not fully reflect grassroots dynamics or long-term historical trends. Despite these limitations, the selected corpus provides a representative overview of recent policy orientations. Future research could expand the scope by incorporating municipal-level data, pre-2015 documents, or mixed sources to enhance the depth and generalizability of policy evaluation.

### 3.2 Coding framework and content analysis

To enable systematic and replicable analysis, the selected policy texts were coded and analyzed using NVivo 14 and ROST-CM6 software. The coding framework was constructed based on three key analytical dimensions: policy tools (X-axis), policy objectives (Y-axis), and policy thematic features (Z-axis), drawing on established literature in public policy analysis (20).

#### 3.2.1 Policy tool dimension (X-axis)

The supply-oriented policy tools of public sports service policies refer to the policy tools directly provided by the government. This dimension classified policy instruments into supply-oriented tools (project support, talent cultivation, infrastructure development, financial subsidies), demand-oriented tools (policy incentives, government procurement, market shaping), and environmental tools (regulatory measures, tax incentives, strategic planning). To enhance interpretability, the data was tabular to highlight the changes in tool usage over time and to detect potential inflection points. Special attention should be paid to the distribution around 2020, which corresponds to the policy strengthening around the “Healthy China 2030” agenda. To ensure consistency and accuracy, all data have undergone cross-validation. This analysis aims to reveal the structural transformation in governance logic and the relative emphasis on different types of tools across policy cycles.

#### 3.2.2 Policy objective dimension (Y-axis)

Policy objectives represent the ultimate aims, requirements, and expected outcomes that guide policy implementation. The scientific determination of public policy objectives depends on a thorough analysis of underlying values, as well as the balance between objectivity and normative expectations. This approach helps to ensure that policy goals are both rational and actionable, thus enhancing the overall quality and effectiveness of public decision-making. Building on this perspective, the present study systematically reviews the content of public sports service policy texts and distills policy objectives into two principal domains: “public interest” and the “Healthy China” initiative. Specifically, the “public interest” domain encompasses three key dimensions: equity, economic benefit, and People-oriented, while the “Healthy China” domain comprises objectives related to improving national physical fitness and advancing sports industry development.

#### 3.2.3 Policy thematic feature dimension (Z-axis)

Given the considerable length and complexity of public sports service policy documents, the identification of key topics and focal areas relies heavily on the analysis of thematic keywords. Keyword frequency analysis is recognized as a fundamental method in content analysis, enabling researchers to objectively capture the central themes and policy priorities embedded in the text. In this study, the ROST-CM6 software was employed to segment and analyze 76 public sports service policy documents, extracting high-frequency keywords to reveal dominant topics. Furthermore, a semantic network was constructed among these keywords, providing deeper insights into the thematic structure and interrelationships that characterize public sports service policy discourse.

**Figure 1:**
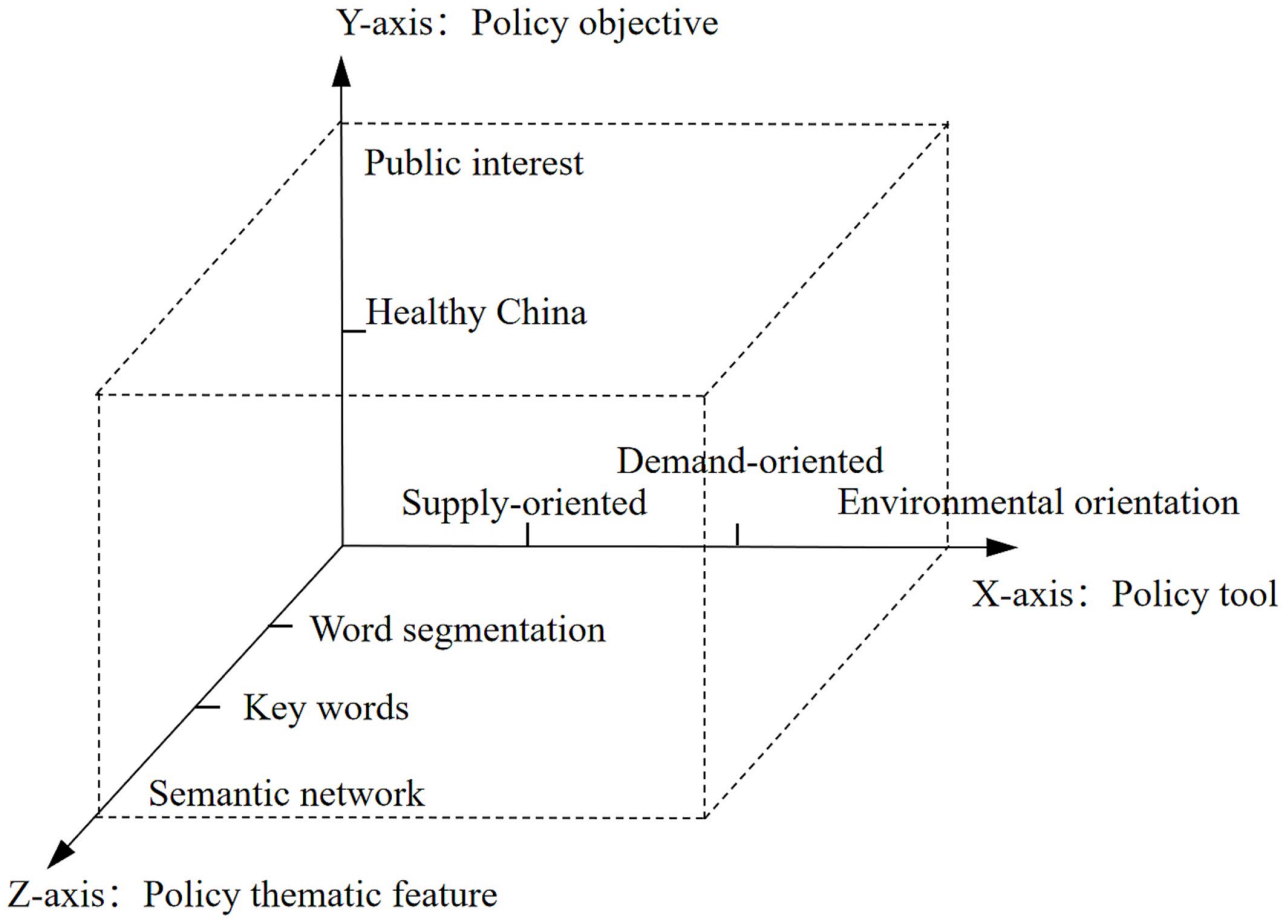
Three-dimensional analysis framework for public sports service policy texts.

Based on the contents of the X dimension, Y dimension and Z dimension mentioned above, Figure 1 constructs a three-dimensional analysis framework for the policy text of public sports services.

### 3.3 Encoding of policy text content

By encoding the collected policy texts, the usage of supply-oriented, demand-oriented and environment-oriented policy tools in public sports service policies can be presented intuitively, and at the same time, the goals that the government’s public sports service work aims to achieve in the next decade can be known relatively accurately. Each clause in the public sports service policy text is encoded with the software Nvivo14. Clauses in the policy text that do not involve the use of policy tools are not encoded in this article. If the same clause involves two or more policy tools, it is encoded repeatedly. Table 2 shows the coding of 76 public sports service policy texts, with 1,598 reference points obtained in the policy tool dimension and 1,461 reference points obtained in the policy objective dimension.

**Table 2:** Text content coding for public sports service policies.

Spindle coding	Open coding	Node	Source of materials	Reference point
Policy tool dimension (1598)	Supply-oriented	Project Support	48	172
		Talent Cultivation	32	78
		Infrastructure Construction	55	269
		Financial Subsidies	34	63
		Popular Science Publicity	44	119
	Demand-oriented	Policy Support	30	71
		Government Purchase	29	124
		Market Shaping	38	136
	Environmental Orientation	Regulatory Control	66	235
		Tax Incentives	8	12
		Goal Planning	57	272
		Policy Support	25	46
Policy objective dimension (1461)	Healthy China	National Physical Fitness	65	397
		Industrial Development	65	496
	Public Interest	Fairness	64	237
		Economy	31	91
		People-oriented	54	236

### 3.4 Quality assurance and reliability

To enhance the reliability and validity of the analysis, coding was conducted independently by two researchers with expertise in policy analysis and public management. Discrepancies were resolved through iterative discussion and, where necessary, consultation with senior experts. The coding results were also benchmarked against existing classification standards from previous literature (22, 23). This multi-step process ensured both the accuracy and replicability of the findings, supporting robust subsequent analysis.

## 4 Quantitative analysis results of public sports service policy texts

### 4.1 X-axis: frequency analysis of policy tool dimension

After systematic coding and categorization of the policy texts, the frequency distribution of policy tools was calculated (For details, please refer to the Supplementary materials). The results indicate a clear hierarchy in the use of policy instruments: supply-oriented policy tools are the most prevalent, followed by environmental tools, with demand-side tools being the least utilized. Out of 1,597 total reference points identified across all analyzed policy clauses, supply-oriented tools account for 701 instances (43.9%), environmental tools for 565 (35.4%), and demand-oriented tools for 331 (20.7%). This distribution reflects the long-standing tradition in China’s public sports policy of prioritizing direct government intervention and provision, while gradually expanding the role of regulation and environmental incentives, and only more recently incorporating demand stimulation through market and social forces.

To gain a more nuanced understanding, the study further analyzed the specific components within each policy tool category, examining the frequency and contextual usage of their constituent elements.

#### 4.1.1 Supply-oriented policy tools

Within the supply-oriented tools, infrastructure development emerges as the most frequently referenced component, with 269 coding references. This high proportion underscores the central role of physical facility construction in supporting the expansion and improvement of public sports services, aligning with China’s ongoing efforts to close the gap in sports infrastructure, especially in under-resourced regions. The importance of project-based support is also evident, accounting for 172 references. These projects often target particular populations or regional characteristics, contributing to the overall diversity and accessibility of services.

Science popularization, with 119 references, serves a critical function in promoting health awareness and public knowledge about fitness, thus laying the groundwork for societal participation in the “Healthy China” initiative. The focus on talent cultivation recorded 78 times, highlights ongoing efforts to develop a professional workforce capable of sustaining the long-term advancement of the sports industry. While direct financial subsidies appear less frequently (63 references), they nonetheless play a crucial role in offsetting operational costs and incentivizing service providers, especially in the context of free or low-cost facility access for the public. Collectively, the prominence of these supply-side mechanisms demonstrates the state’s strategic commitment to building robust material and human resource foundations for public sports service delivery.

#### 4.1.2 Demand-oriented policy tools

In the realm of demand-oriented policy tools, the data reveal a different pattern. Market-shaping measures represent the largest share (136 references), signaling a gradual policy shift toward activating consumer demand and supporting the growth of sports-related service industries. This reflects the policy agenda’s increasing emphasis on integrating public sports with broader sectors such as health, senior care, culture, and tourism, an approach considered essential for fostering innovative and sustainable service models.

Government procurement, with 124 references, highlights the state’s ongoing reliance on direct purchasing as a mechanism to improve the efficiency and coverage of public sports services, promoting standardization, equity, and professionalization through contracting. Policy incentives, although somewhat less frequently cited (71 references), remain an important tool for guiding the sector’s development, particularly through financial support, subsidies, and loan facilitation. Overall, while demand-side tools remain less dominant than supply or environmental tools, their growing presence in policy discourse reflects a recognition of the need to diversify service provision models and encourage greater societal engagement.

#### 4.1.3 Environmental policy tools

Environmental policy tools encompass regulatory frameworks, tax incentives, strategic planning, and policy support measures that together shape the broader institutional context of public sports service delivery. Among these, strategic goal-setting and planning have the highest frequency (272 references), suggesting that policymakers place substantial emphasis on articulating ambitious targets, such as expanding facility coverage, increasing participation rates, and enhancing industry output. While such objectives are critical for mobilizing resources and guiding implementation, there is also a risk of overly prescriptive or idealistic planning, which may sometimes lack actionable pathways or adaptability to local contexts.

Legal and regulatory measures are cited 235 times, reflecting the growing importance of law-based governance in public service management. The establishment and enforcement of standards, accountability systems, and contractual mechanisms are increasingly used to ensure transparency, fairness, and compliance in the execution of public sports projects. By contrast, direct policy support (46 references) and tax incentives (12 references) are much less prominent. This suggests that, despite their recognized value in promoting sectoral growth and reducing cost barriers, these tools have yet to be fully leveraged within the existing policy framework. The limited use of financial incentives and targeted support mechanisms may represent an area for further improvement, especially in enhancing the economic viability and inclusivity of public sports service provision.

Overall, the analysis of the policy tool dimension reveals a predominant reliance on traditional government-led, supply-oriented interventions, a strong but sometimes top-down orientation in planning and regulation, and an emerging yet still limited focus on demand-side stimulation and targeted economic incentives. These findings underscore the evolving but still unbalanced nature of public sports service policy in China, highlighting both the achievements and remaining challenges in the quest for high-quality, equitable, and sustainable sports service provision.

#### 4.1.4 Temporal evolution of policy tool usage

From 2015 to 2025, the use of policy tools in China’s public sports service sector has undergone a notable structural shift (Table 3). Supply-oriented tools remained dominant throughout the period but showed a gradual decline, decreasing from 136 instances in 2019–2020 to 111 in 2023–2025. This trend indicates a move away from direct government provision toward more collaborative and diversified approaches. Demand-oriented tools experienced a significant rise, increasing from only 28 in 2015–2016 to 89 in 2023–2025. This upward trend reflects growing emphasis on stimulating public participation, outsourcing services, and incentivizing social actors, signaling a transition from provider-driven to user-responsive policy design. Environmental tools, though fewer in number, showed steady growth. These tools primarily supported regulatory frameworks, strategic planning, and performance evaluation, reinforcing institutional support for systematization and quality assurance.

**Table 3:** Temporal trends in policy tool usage (two-year bins, 2015–2025; counts).

Tool type	2015–2016	2017–2018	2019–2020	2021–2022	2023–2024	2025 YTD*	Row total
Supply-oriented	109	119	136	129	97	111	701
Demand-oriented	28	41	69	68	89	36	331
Environmental Orientation	82	91	106	104	86	96	565
Total references	219	251	311	301	272	243	1,597

*2025 YTD reflects policies released/implemented up to the time of data collection.

A turning point emerged around 2019 when the growth rate of supply tools slowed, while the adoption of demand and environmental tools accelerated. This shift coincided with the implementation of the Healthy China initiative and the broader digital transformation of public services, reflecting a more balanced and adaptive policy mix.

Overall, the evolving distribution of policy tools illustrates a transformation in governance logic. The public sports service system is gradually moving from centralized control to multi-actor collaboration, with a stronger focus on flexibility, precision, and long-term sustainability.

### 4.2 Y-axis: frequency analysis of policy objective dimension

**Figure 2:**
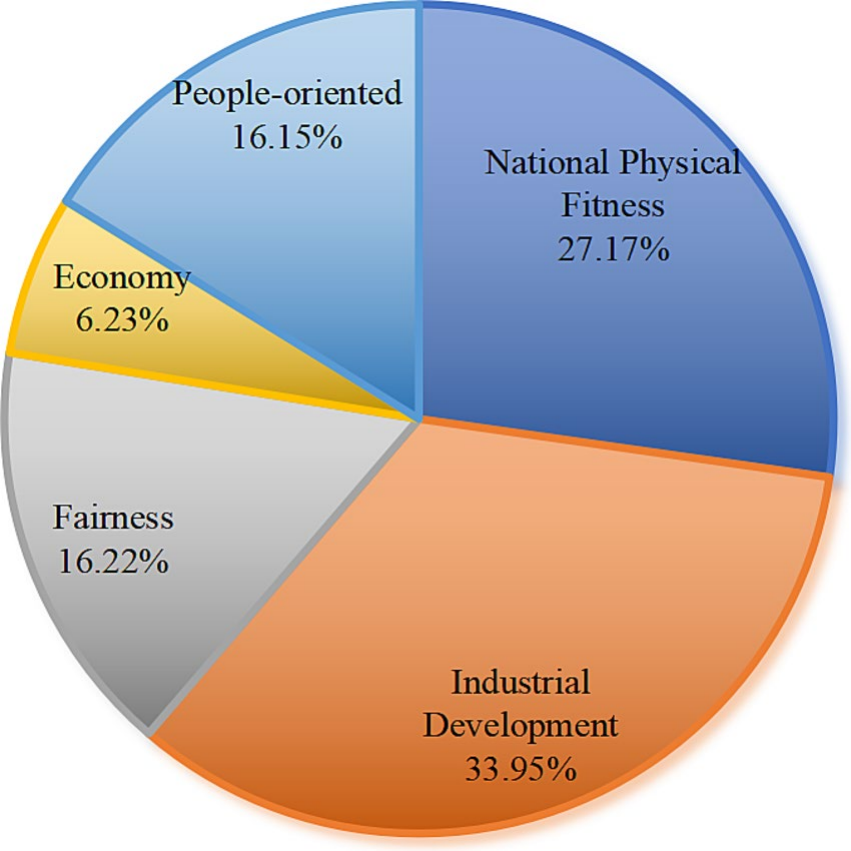
Coding statistics of policy objective dimensions.

Building on the quantitative coding of policy objectives, this study provides an in-depth analysis of the guiding priorities in China’s public sports service policy system, as depicted in Figure 2. The objectives are grouped into two main domains, “Healthy China” and “public interest,” each further divided into concrete sub-objectives: industrial development, national physical fitness, equity, people-centeredness, and economic benefit. This multi-layered structure not only reflects the evolving strategic direction of China’s sports policies but also offers a basis for comparison with leading international policy frameworks.

Industrial development emerges as the most prominent policy objective, accounting for 33.95% of all coded references (496 references). This strong focus highlights China’s strategic vision to cultivate the sports industry as a driver of economic modernization and as a core platform for high-quality public service provision. Notably, the National Fitness Program (2021–2025) sets clear targets for expanding the sports industry’s scale, supporting the growth of sports service enterprises, and promoting “sports + tourism” pilot zones and industry clusters. These efforts are aligned with international trends, for example, the European Union’s White Paper on Sport (24) emphasizes the economic value of sport and its contribution to innovation and employment across member states.

Closely following, national physical fitness represents 27.17% of coded objectives (397 references), underpinning the “Healthy China 2030” national strategy. A hallmark of Chinese policy in this area is the implementation of “15-min fitness circles,” designed to ensure the majority of residents can access sports facilities within a short walking distance. This goal is supported by comprehensive public participation campaigns and a robust school-based fitness program. Similar nationwide physical activity targets are seen in Japan’s Sport Basic Plan (25), which stresses equal access and lifelong engagement in physical activity for all population groups. By systematically raising the physical fitness level of the population, these measures contribute directly to the long-term goals of disease prevention, healthy aging, and improved quality of life.

Social fairness and inclusivity are embedded in the dual objectives of equity (16.22%, 237 references) and people-centeredness (16.15%, 236 references). Policies such as the Notice on Further Promoting the Construction of Public Sports Facilities and the National Fitness Program (2021–2025) earmark investments for rural and marginalized communities, mandate the creation of barrier-free facilities for persons with disabilities, and require targeted support for children and older adults. This approach is consistent with the UK’s Sporting Future strategy (26), which aims to address disparities in sports participation and promote a more physically active and inclusive society. These objectives in China are operationalized through legal protections, needs-based facility allocation, and the establishment of diverse participation mechanisms, all intended to reduce inequalities and enhance satisfaction for all citizens.

While economic benefit is the least cited among the major objectives (6.23%, 91 references), it plays a crucial supporting role. Policies such as the Action Plan for Further Promoting Sports Consumption (27) directly address the economic potential of sports, promoting the development of sports goods markets, stimulating sports tourism, and encouraging private sector investment in service innovation. This reflects a growing recognition of the sector’s value in driving consumption and local economic vitality: an orientation also seen in Australia’s “Sport 2030 - National Sport Plan” (28), which frames sport as an engine of national productivity and export growth.

Taken together, these results reveal that China’s public sports service policies pursue a multidimensional balance among economic modernization, population health, and social equity, combining ambitious industry and fitness targets with explicit commitments to fairness and inclusivity. The integration of concrete policy initiatives and global best practices suggests a sophisticated and responsive policy system. However, the lower coding frequency for economic objectives points to the need for more robust mechanisms for assessing and optimizing the economic impacts of sports policy, in line with advanced international standards.

### 4.3 Z-axis: analysis of policy thematic features

#### 4.3.1 Analysis of high-frequency keywords

Utilizing the ROST-CM6 content analysis software, this study extracted the most frequently occurring keywords from the public sports service policy documents to systematically capture the core thematic features embedded within policy discourse. Given the substantial length and diversity of the policy texts, the analysis focused on the top 60 high-frequency keywords, as shown in Table 4. This approach enables a nuanced understanding of policy priorities, strategic orientations, and evolving discursive trends across the public sports service sector.

**Table 4:** High-frequency key words in public sports service policy texts.

Key word 1	Word frequency1	Key word 2	Word frequency2	Key word 3	Word frequency3	Key word 4	Word frequency4
Health	605	Establish	175	Subject	117	State Affairs	93
Construction	588	Promote	170	Technology	114	Sound	93
Development	574	Public welfare	148	Policy	113	Teenagers	92
Public	525	System	147	Field	112	Science	91
National fitness	484	Formulate	146	Innovation	111	Knowledge	91
Fitness	478	Implementation	145	Regulations	109	Conditions	90
Society	470	Level	142	Feature	108	Community	90
Country	403	Unit	138	Safety	105	Healthy China	90
Government	371	Reform	136	Sufficient	104	File	89
Facilities	313	Education	132	Give full play	99	Region	89
Standard	302	Engineering	131	Key point	98	Power	83
The people	244	Guarantee	129	Effective	97	Funds	82
The masses	236	Economy	126	Plan	95	Local	80
Culture	201	Resources	123	Realize	95	According to	80
Nationwide	185	Participate	120	Function	94	Consumption	80

At the highest frequency tier, keywords such as “Health” (605 times), “Construction” (588), “Development” (574), “Public” (525), and “National fitness” (484) stand out as the dominant concepts. Their prevalence reflects the central strategic narrative of public sports service policy namely, to improve population health, accelerate the construction and modernization of sports infrastructure, promote comprehensive sector development, and uphold the value of public service for all citizens. The prominence of “National fitness” and “Fitness” (478) highlights the policy system’s deep commitment to advancing the “Healthy China” initiative and embedding physical activity as a cultural and social norm.

Other highly ranked terms, such as “Society” (470), “Nation” (403), “Government” (371), “Facilities” (313), and “Standard” (302), signal the multifaceted, system-level approach adopted in policy formulation and implementation. The frequency of “Society” and “Government” reflects an interplay between state leadership and social participation, while “Facilities” and “Standard” underscore the emphasis on equitable, high-quality resource allocation and institutionalized performance metrics. The term “The people” (244) demonstrates the ongoing policy focus on people-centeredness, public welfare, and citizen satisfaction as foundational principles.

Further down the list, keywords such as “Culture” (201), “Participation” (185), “Establish” (175), “Promote” (170), “Public welfare” (148), “System” (147), “Formulate” (146), and “Implementation” (145) illuminate the operational pathways through which policies are enacted and sustained. The recurrence of terms like “Public welfare,” “Guarantee” (129), and “Resources” (123) highlights policy support for vulnerable groups, resource mobilization, and the institutional guarantee of universal service. “Reform” (136), “Education” (132), and “Engineering” (131) point to ongoing efforts in system modernization, talent development, and technical capacity building. Keywords that describe operational functions and policy mechanisms, such as “Subject,” “Technology,” “Policy,” “Field,” “Innovation,” “Regulations,” “Feature,” “Safety,” “Sufficient,” “Key point,” “Effective,” “Plan,” “Realize,” and “Function,” reveal a strong focus on pragmatic implementation, innovation, and continual improvement. The high frequency of “Technology” and “Innovation” indicates the sector’s increasing orientation toward digitalization and new models of sports service delivery. “Safety” and “Regulations” indicate concern for risk management and governance in both service operations and facility construction. Additionally, context-specific terms, like “State Affairs,” “Teenagers,” “Science,” “Knowledge,” “Community,” “Healthy China,” “File,” “Region,” “Funds,” “Local,” “According to,” and “Consumption,” emphasize both the granularity and the complexity of the policy landscape. For instance, “Teenagers” and “Community” point to the focus on targeted population segments and grassroots-level participation, while “Funds” and “Consumption” reference the economic and financial dimensions of policy execution.

Collectively, the breadth and distribution of high-frequency keywords paint a portrait of a dynamic, multidimensional policy system that combines visionary goals (“Health,” “Development,” “National fitness”) with robust institutional mechanisms (“Government,” “Standard,” “Implementation”), a commitment to public value (“The people,” “Public welfare,” “Participation”), and an evolving toolbox for reform, innovation, and evidence-based management. The recurring attention to “Society,” “Community,” and “Culture” underscores the aim to achieve both social integration and cultural enrichment through sports, while the presence of economic and technical terms suggests a trend toward modernization and sustainability. The thematic keyword analysis demonstrates that China’s public sports service policies are not only strategically ambitious but also operationally detailed, addressing the needs of diverse stakeholders and evolving in line with global trends in public health and social governance.

#### 4.3.2 Semantic network analysis of thematic keywords

**Figure 3:**
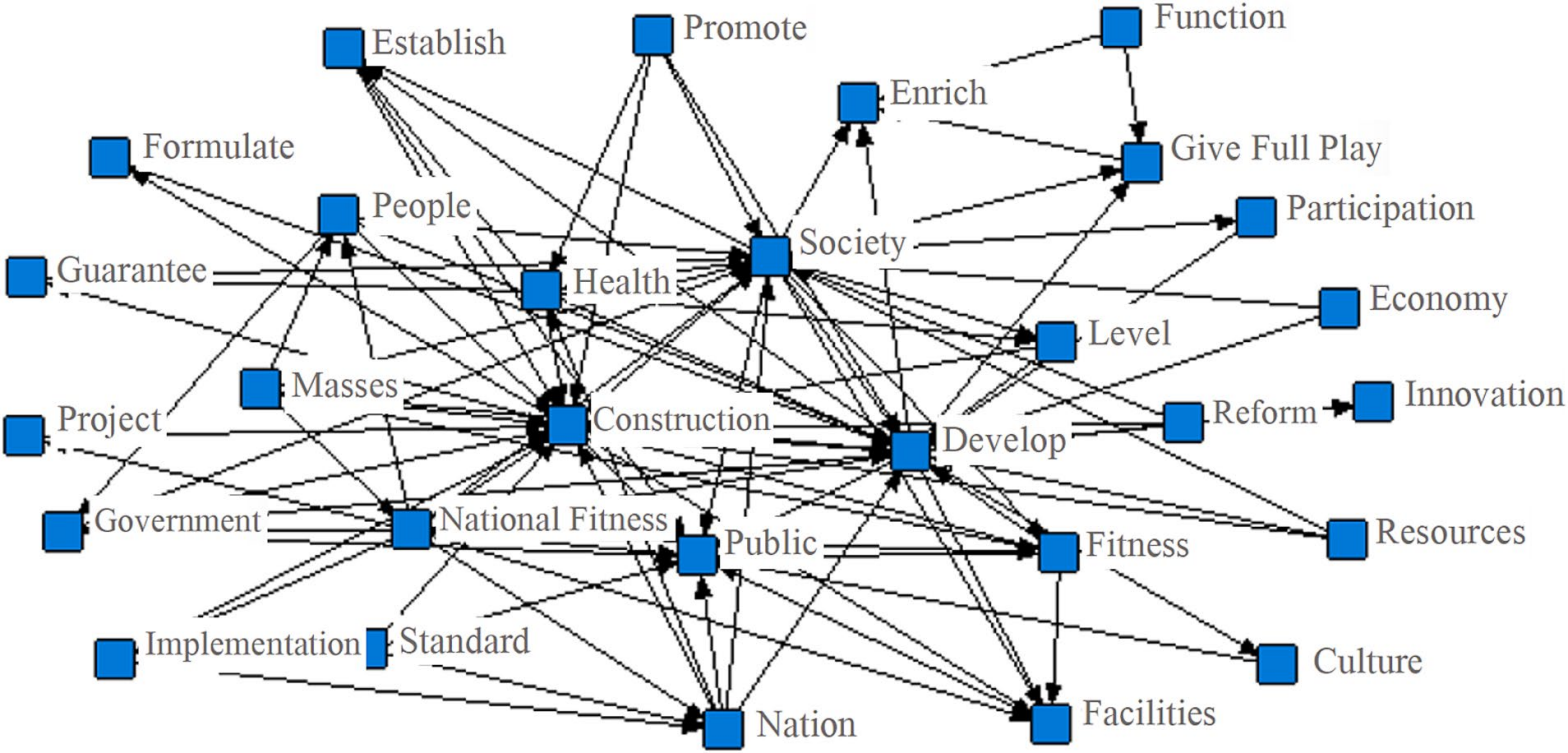
Semantic network of subject words in the public sports service policy text.

The application of semantic network analysis to public sports service policy texts offers a vivid and intuitive perspective on the main focal points and thematic priorities embedded in policy discourse. By consolidating the policy corpus into a unified document and leveraging the NetDraw module in ROST-CM6, a comprehensive semantic network of keywords was generated (see Figure 3). This network not only visually delineates the structure of policy attention but also facilitates the exploration of linkages among core and high-frequency terms, thereby providing empirical evidence of policy logic and coordination.

The semantic network (Figure 3) reveals robust interconnections among numerous keywords, with several emerging as central nodes in the overall architecture. “Construction,” “Development,” “Society,” “Public,” and “Health” are at the heart of the network, each serving as both a focal point and a bridge to other policy concepts. The repeated and prominent association of “Construction” and “Development” underscores the government’s ongoing commitment to infrastructure building, facility upgrading, and technological advancement. This reflects not only an aspiration for material expansion but also a policy orientation towards systemic growth and modernization in public sports services.

The central positioning of “Society” and “Public” illustrates the fundamentally collective nature of public sports service policies. These terms highlight a dual orientation: on the one hand, policies are designed to address the needs and demands of the general public; on the other, they seek to harness the power of social participation and community mobilization. This is consistent with the broader policy goal of fostering social harmony, enhancing public cohesion, and ensuring that sports services act as vehicles for public welfare and societal well-being.

Adjacent to the network’s core are keywords such as “People,” “Masses,” “National Fitness,” and “Reform.” Their close proximity signals the deep-rooted commitment to serving citizens and the broader population, a “people-first” ethos that is further embodied in the government’s promotion of national fitness initiatives and health campaigns. The presence of “Reform” also points to the continuous evolution and adaptation of public sports policy in response to changing societal needs and the push for systemic innovation.

Other important nodes, such as “Guarantee,” “Implementation,” “Standard,” “Facilities,” “Resources,” and “Establish” map out the operational and institutional landscape of public sports service provision. The interlinkages among these terms indicate the policy system’s focus on ensuring robust service guarantees, effective policy implementation, comprehensive standardization, resource allocation, and ongoing institutional building. Such a configuration underscores the multi-dimensional strategy employed to drive progress and address emerging challenges in public sports services.

Interestingly, the network also identifies peripheral or relatively isolated nodes, such as “Innovation,” which appears somewhat disconnected from the main semantic clusters. Suggesting that it lacks meaningful conceptual connections with dominant themes in public sports service policy. This marginality may be symptomatic of deeper structural challenges. From an institutional perspective, public sector governance in China is still largely shaped by top-down administrative logics, which often favor stability and compliance over experimentation and flexibility. Consequently, innovative ideas may struggle to gain traction within policy formulation processes. Additionally, funding mechanisms tend to prioritize tangible outputs, such as infrastructure development or service expansion rather than investing in pilot programs, risk-taking, or technology-driven solutions that are inherently uncertain. Finally, the limited involvement of non-governmental stakeholders, such as social organizations, private enterprises, and citizens, restricts opportunities for collaborative innovation. The peripheral position of innovation in the policy discourse thus reveals an underlying tension between traditional governance structures and the emerging need for adaptive, participatory, and future-oriented public service reforms.

This pattern suggests that, while innovation is acknowledged within policy rhetoric, it has not yet been fully integrated into the mainstream discourse or operational pathways of public sports service governance. The relative isolation of “Innovation” may point to underlying institutional inertia, limited policy incentives, or challenges in translating innovative concepts into actionable programs—a gap that warrants further attention in future policy reforms.

Overall, the semantic network analysis not only illuminates the structural coherence and thematic complexity of China’s public sports service policies but also highlights both the strengths and potential areas for improvement. The close integration of construction, development, social engagement, and public value represents a policy system that is ambitious and responsive. At the same time, the comparative marginalization of innovation within the network signals a need for greater emphasis on creativity, experimentation, and knowledge-driven reform as China’s public sports sector continues to evolve.

## 5 Conclusion

This study employs a systematic content analysis approach, constructing an “X–Y–Z” three-dimensional analytical framework to code and interpret 76 representative public sports service policy documents. The study conclusions are as follows: (1) the analysis of the policy tool dimension (X-axis) reveals significant disparities in the frequency of use among different types of policy instruments. Supply-oriented tools are overwhelmingly dominant, reflecting China’s historical reliance on direct government investment and service provision. Environmental tools, which include regulatory, planning, and support measures, are also widely used, underscoring the importance of institutional and legal guarantees in the governance system. By contrast, demand-oriented tools—such as market incentives, public participation initiatives, and consumer-oriented programs—are less frequently employed, indicating room for further policy innovation to mobilize societal forces and meet diverse public needs. (2) With respect to the policy objective dimension (Y-axis), the study differentiates between two key strategic domains: “Healthy China,” encompassing sub-goals of national physical fitness and industrial development, and “public interest,” which comprises equity, economic efficiency, and people-centeredness. Frequency analysis demonstrates clear disparities in policy attention: objectives related to industrial development and population fitness occupy a much larger share of policy references, while economic benefit, equity, and people-centeredness although present receive relatively less systematic emphasis. This pattern highlights the government’s dual strategy of positioning public sports services as both an engine of economic modernization and a foundational pillar for national health improvement. (3) Thematic keyword extraction and semantic network analysis further reinforce these findings. The keywords “Health,” “Construction,” “Development,” and “Public” emerge as the most central themes within the policy corpus, indicating that health improvement, infrastructure expansion, and collective welfare remain at the core of the policy agenda. Notably, the term “Innovation” appears relatively isolated in the semantic network, suggesting that although the rhetoric of innovation is present, its integration into mainstream policy design and operational practice remains limited. This underscores the need for a more proactive approach to fostering creativity, technological advancement, and flexible service models within the public sports sector.

From an international perspective, several countries have developed mature policy frameworks that address similar challenges in public sports governance. For instance, the European Union’s White Paper on Sport emphasizes the economic and social role of sport, calling for multi-actor cooperation and evidence-based policymaking. Japan’s Sport Basic Plan focuses on promoting equal access to physical activity through lifelong participation, echoing China’s “15-min fitness circles” initiative. Australia’s Sport 2030 Strategy integrates sport with national productivity, public health, and community development goals, placing strong emphasis on innovation, digital tools, and public-private collaboration. Table 5 compares the policy tool structures and governance models of public sports services across China, the UK, the EU, and Japan, highlighting China’s ongoing transition from a supply-driven system toward more diversified and participatory approaches. Drawing from these cases, China’s public sports service system could further benefit from a more diversified policy toolkit, greater integration of demand-side instruments, and stronger mechanisms for stimulating innovation and social participation. These international comparisons not only validate the study’s findings but also provide feasible directions for enhancing the design and implementation of China’s public sports policies.

**Table 5:** Comparative analysis of public sports service policy tools across China, the UK, the EU, and Japan.

Dimension	China	United Kingdom	European Union	Japan
Policy tool structure	Supply-driven; supported by demand and environment tools	Demand-oriented (marketization and outsourcing)	Environment-dominant (legal frameworks and strategic planning)	Community-oriented (autonomous governance tools)
Service providers	Government-led with emerging social participation	Non-profits, local councils, private enterprises	Multistakeholder coordination across member states	Neighborhood associations + local sports clubs
Legal and institutional guarantees	Basic legal support	Health and sports laws + regulatory performance systems	Binding regulations + soft law instruments	Legislation grounded in “lifetime sports” philosophy
Policy evolution trend	Transitioning toward co-governance and collaborative creation	From performance-driven to balanced community-based models	From strategic orientation to social resilience	From traditional clubs to digital and intergenerational integration

Given the findings above, several recommendations are put forward to enhance the design, implementation, and long-term impact of public sports service policies in China. (1) Regarding the selection and integration of policy tools, it is essential to fully harness the power of market mechanisms, while pursuing a coordinated and flexible mix of demand-side, supply-side, and environmental instruments. As China continues to advance its supply-side structural reforms and the “dual circulation” economic model, there is a strategic opportunity to diversify governance approaches and optimize the policy toolkit. Demand-oriented tools, such as incentives for public participation, awareness campaigns, and health literacy programs, should be expanded to stimulate latent demand and support behavioral change across population segments. Supply-side measures should focus not only on increasing investment in sports facilities and improving service quality, but also on promoting the development of professional talent and innovative service platforms. Environmental tools, particularly those that improve public space, standardize legal protections, and foster intersectoral collaboration are vital for ensuring equal access and safeguarding user rights. Critically, policy effectiveness will depend on the synergistic application of these tools, underpinned by scientific evaluation, periodic adjustment, and robust public engagement and feedback mechanisms. (2) With respect to policy objective setting, a multi-dimensional and dynamic approach should be adopted. Policymakers should integrate the core imperatives of the “Healthy China” strategy, social equity, citizen rights, sports industry development, and national image enhancement within a holistic policy framework. The primary aim must remain the improvement of public health and the prevention of chronic diseases, achievable through widespread health education and the promotion of evidence-based exercise behaviors. At the same time, policy must address imbalances in the allocation of sports resources between urban and rural areas, paying special attention to the needs of remote, rural, and marginalized groups. Accelerating the development of the sports industry and piloting innovative service models, such as public-private partnerships and “Internet+Sports” can increase accessibility, promote integration with sports tourism, and catalyze regional economic development. (3) Policies should seek to enhance both elite sports performance and grassroots participation, fostering international exchanges that strengthen national competitiveness and image on the world stage. Achieving these goals requires a careful balance between sometimes competing objectives, continuous scientific assessment, adaptive policy adjustment, and broad stakeholder consultation. By integrating multi-objective demands and maintaining a commitment to evidence-based decision-making, policymakers can ensure that public sports service reforms are both rational and effective, ultimately driving high-quality, sustainable development in the sector.

### 5.1 Limitations and future research

This study has several limitations. First, it focuses solely on the content of policy documents without examining their actual implementation or outcomes, limiting its ability to assess real-world effectiveness. Second, despite efforts to ensure coding reliability, the manual classification of policy tools and objectives inevitably involves some subjectivity. Third, the lack of longitudinal outcome indicators, such as public health data or participation rates precludes deeper analysis of policy impact over time. Lastly, software-assisted content analysis tools like ROSTCM6, while powerful, may overlook nuanced meanings or institutional contexts embedded in policy language. Future research could incorporate stakeholder interviews, field data, or cross-national implementation comparisons to triangulate and enrich findings.

## Data Availability

The original contributions presented in the study are included in the article/Supplementary material, further inquiries can be directed to the corresponding author.
