# Supplementary material for: Policy tools, objectives, and governance in China’s public sports services: an X–Y–Z framework analysis of national and provincial policies
Source: Front Public Health. 2025 Oct 28;13:1692238. doi: 10.3389/fpubh.2025.1692238 (PMC12602396; doi:10.3389/fpubh.2025.1692238)
Supplement: Supplementary file 1 [file Table_1.DOCX]

**Supplementary Materials**

Table1 Statistics on the encoding frequency of policy tool dimensions

| **Parent node** | **Child node** | **Source of materials** | **Reference point** | **Examples of reference points** | **Coverage percentage (%)** | **Classified total** |
| --- | --- | --- | --- | --- | --- | --- |
| Supply-oriented | Project Support | 48 | 172 | We should vigorously develop sports that are popular among the masses and encourage the development of characteristic sports that suit different groups of people and regional features. | 10.8 | 701  （43.9%） |
|  | Talent Cultivation | 32 | 78 | Strengthen the cultivation of talents in the sports industry and form a high-level talent cultivation system that effectively supports the development of the sports industry. | 4.9 |  |
|  | Infrastructure Construction | 55 | 269 | In line with the local sports and fitness venue and facility construction plan, ensure the land for equipment installation, allocate matching funds, and guarantee the quality of venue and facility construction that is compatible with the equipment. | 16.8 |  |
|  | Financial Subsidies | 34 | 63 | In line with the local sports and fitness venue and facility construction plan, ensure the land for equipment installation, allocate matching funds, and guarantee the quality of venue and facility construction that is compatible with the equipment. | 3.9 |  |
|  | Popular Science Publicity | 44 | 119 | Make full use of government bulletins, government websites, radio, television and new media platforms, etc. to strengthen the publicity and interpretation of basic public service standards. | 7.5 |  |
| Demand-oriented | Policy Support | 30 | 71 | Improve the public finance investment mechanism for sports, and provide support through measures such as matching subsidies and loan interest subsidies in accordance with the development needs of the sports service industry. | 4.4 | 331  （20.7%） |
|  | Government Purchase | 29 | 124 | The government purchases public cultural and sports services with the aim of enhancing the supply efficiency of such services, striving to achieve standardized, equalized and socialized development of public cultural and sports services. | 7.8 |  |
|  | Market Shaping | 38 | 136 | Vigorously develop the sports service industry, actively guide mass sports consumption, expand the scope of public sports services, and promote the common development of the sports industry with health, elderly care, culture, tourism and other industries. | 8.5 |  |
| Environmental orientation | Regulatory Control | 66 | 235 | In strict accordance with the laws and regulations on construction management such as the project legal person responsibility system, the bidding and tendering system, the engineering supervision system and the contract management system, we should earnestly carry out the project construction and management work. | 14.7 | 565  （35.4%） |
|  | Tax Incentives | 8 | 12 | Improve tax and fee policies. For the self-used properties and land of large sports venues, if they meet the conditions stipulated by tax laws and regulations, they can enjoy preferential policies on property tax and urban land use tax. | 0.8 |  |
|  | Goal Planning | 57 | 272 | A city-wide public sports service system with more widespread facilities, more complete organization, more diverse activities, better services and greater public satisfaction has been initially established, achieving equalization of public sports services. | 17 |  |
|  | Policy Support | 25 | 46 | Improve policy support. The provincial sports industry development special funds and the subsidy funds for free and low-cost opening of sports venues will provide support to sports service complexes that meet the conditions. | 2.9 |  |
| Total | | | | |  | 1597 |
